# Supplementary material for: NADPH oxidase-mediated redox signaling promotes oxidative stress resistance and longevity through memo-1 in C. elegans
Source: eLife. 2017 Jan 13;6:e19493. doi: 10.7554/eLife.19493 (PMC5235354; doi:10.7554/eLife.19493)
Supplement: Figure 2—source data 1. — DOI: http://dx.doi.org/10.7554/eLife.19493.007 [file elife-19493-fig2-data1.docx]

| Gene | Function | SKN-1 binding site | Reference | SKN-1::GFP ChIP signal | Reference | confirmed by endogenous  SKN-1 ChIP | Reference |
| --- | --- | --- | --- | --- | --- | --- | --- |
| *gcs-1* | gamma-glutamine cysteine synthetase | 3 | (An & Blackwell 2003)  (Oliveira et al. 2009) | low | Niu et al., 2011) | yes | (Robida-Stubbs et al. 2012) |
| *gst-1* | Glutathione S-transferase | 1 | (Oliveira et al. 2009) | high | Niu et al., 2011) |  |  |
| *gst-4* | Glutathione S-transferase | 3 | (An & Blackwell 2003)  (Oliveira et al. 2009) | high | Niu et al., 2011) | yes | (Robida-Stubbs et al. 2012)  (Glover-Cutter et al. 2013) |
| *gst-5* | Glutathione S-transferase | 4 | (An & Blackwell 2003)  (Oliveira et al. 2009) | high | Niu et al., 2011) |  |  |
| *gst-38* | Glutathione S-transferase | 2 | (An & Blackwell 2003)  (Oliveira et al. 2009) | low | modencode.org |  |  |
| *ugt-8* | UDP glycosyltransferase | 1 |  | low | modencode.org |  |  |
| *ugt-25* | UDP glycosyltransferase | 3 |  | low | modencode.org |  |  |
| *ugt-38* | UDP glycosyltransferase | 2 |  | low | modencode.org |  |  |

**Figure 2- source data 1. Oxidative stress response genes upregulated by loss of *memo-1* are transcriptional targets of SKN-1.**
