# Supplementary material for: NADPH oxidase-mediated redox signaling promotes oxidative stress resistance and longevity through memo-1 in C. elegans
Source: eLife. 2017 Jan 13;6:e19493. doi: 10.7554/eLife.19493 (PMC5235354; doi:10.7554/eLife.19493)
Supplement: Supplementary file 1. — DOI: http://dx.doi.org/10.7554/eLife.19493.015 [file elife-19493-supp1.docx]

**Supplementary File 1. Loss of *memo-1* increases adult lifespan**

| Strain / **RNAi** | Mean lifespan ± S.E.M.  [Days] | 75^th^ percentile  [Days] | N dead/ Initial N | % mean lifespan change to control | P-value (log-rank) vs. control | Figure |
| --- | --- | --- | --- | --- | --- | --- |
| **Trials without FuDR** | | | | | | |
| Trial of *memo-1(gk345)* mutants at 20°C (no FuDR) | | | | | | |
| wild type (N2) | 15.4 ± 0.6 | 17 | 85/100 |  |  |  |
| *memo-1(gk345)* mutants | 20.0 ± 0.5 | 22 | 78/99 | +30 | <0.0001 |  |
| *P*-value and % mean lifespan change are relative to wild type (N2) | | | | | | |
| Trial of *memo-1(gk345)* mutants at 20°C (no FuDR) | | | | | | |
| wild type (N2) | 17.3 ± 0.6 | 21 | 77/101 |  |  | Fig. 1C |
| *memo-1(gk345)* mutants | 21.9 ± 0.6 | 25 | 69/103 | +27 | <0.0001 | Fig. 1C |
| *P*-value and % mean lifespan change are relative to wild type (N2) | | | | | | |
| Trial of *memo-1(gk345)* mutants at 25°C (no FuDR) | | | | | | |
| wild type (N2) | 11.0 ± 0.4 | 14 | 72/91 |  |  |  |
| *memo-1(gk345)* mutants | 15.2 ± 0.4 | 18 | 92/101 | +38 | <0.0001 |  |
| *P*-value and % mean lifespan change are relative to wild type (N2) | | | | | | |
| Trial of *memo-1(gk345)* mutants at 25°C (no FuDR) | | | | | | |
| wild type (N2) | 10.2 ± 0.3 | 13 | 88/100 |  |  |  |
| *memo-1(gk345)* mutants | 13.5 ± 0.4 | 15 | 79/100 | +32 | <0.0001 |  |
| *P*-value and % mean lifespan change are relative to wild type (N2) | | | | | | |
| Trial of *memo-1(gk345)* mutants at 25°C (no FuDR) | | | | | | |
| wild type (N2) | 11.5 ± 0.4 | 15 | 79/100 |  |  |  |
| *memo-1(gk345)* mutants | 15.6 ± 0.4 | 17 | 78/98 | +36 | <0.0001 |  |
| *P*-value and % mean lifespan change are relative to wild type (N2) | | | | | | |
| Trial of *memo-1(gk345)* mutants at 25°C (no FuDR) | | | | | | |
| wild type (N2) | 9.9 ± 0.3 | 11 | 94/103 |  |  |  |
| *memo-1(gk345)* mutants | 13.6 ± 0.4 | 15 | 84/112 | +37 | <0.0001 |  |
| *P*-value and % mean lifespan change are relative to wild type (N2) | | | | | | |
| **Trials with FuDR** | | | | | | |
| Trial of *memo-1(gk345)* mutants at 20°C (with FuDR) | | | | | | |
| wild type (N2) | 20.5 ± 0.5 | 25 | 94/110 |  |  |  |
| *memo-1(gk345)* mutants | 22.1 ± 0.4 | 25 | 108/128 | +8 | 0.0104 |  |
| *P*-value and % mean lifespan change are relative to wild type (N2) | | | | | | |
| Trial of *memo-1(gk345)* mutants at 20°C (with FuDR) | | | | | | |
| wild type (N2) | 15.6 ± 0.4 | 18 | 104/130 |  |  |  |
| *memo-1(gk345)* mutants | 19.6 ± 0.5 | 25 | 107/127 | +26 | <0.0001 |  |
| *P*-value and % mean lifespan change are relative to wild type (N2) | | | | | | |
| Trial of *memo-1(gk345)* mutants at 20°C (with FuDR) | | | | | | |
| wild type (N2) | 23.6 ± 0.4 | 28 | 149/162 |  |  |  |
| *memo-1(gk345)* mutants | 25.6 ± 0.4 | 28 | 132/149 | +8 | 0.0009 |  |
| *P*-value and % mean lifespan change are relative to wild type (N2) | | | | | | |
| Trial of *memo-1(gk345)* mutants on heat-killed OP50 bacteria at 20°C (with FuDR) | | | | | | |
| wild type (N2) | 25.7 ± 0.3 | 28 | 94/108 |  |  |  |
| *memo-1(gk345)* mutants | 31.4 ± 0.4 | 35 | 139/155 | +22 | <0.0001 |  |
| *P*-value and % mean lifespan change are relative to wild type (N2) | | | | | | |
| Trial of *memo-*1 knock down by RNAi starting at the first day of adulthood at 20°C (with FuDR) | | | | | | |
| *rrf-3(pk1426)* **L4440 (control)** | 20.4 ± 0.3 | 22 | 133/149 |  |  | Fig. 1D |
| *rrf-3(pk1426)* ***memo-1(RNAi#1)*** | 24.1 ± 0.4 | 26 | 130/145 | +18 | <0.0001 | Fig. 1D |
| *rrf-3(pk1426)* ***memo-1(RNAi#2)*** | 21.9 ± 0.3 | 25 | 155/174 | +7 | 0.0001 |  |
| *P*-value and % mean lifespan change are relative to *rrf-3(pk1426)* RNAi L4440 | | | | | | |
| Trial of *memo-*1 knock down by RNAi starting at the first day of adulthood at 20°C (with FuDR) | | | | | | |
| *rrf-3(pk1426)* **L4440 (control)** | 18.1 ± 0.4 | 22 | 134/151 |  |  |  |
| *rrf-3(pk1426)* ***memo-1(RNAi#1)*** | 21.7 ± 0.4 | 25 | 117/140 | +20 | <0.0001 |  |
| *rrf-3(pk1426)* ***memo-1(RNAi#2)*** | 19.3 ± 0.4 | 22 | 82/114 | +7 | 0.0867 |  |
| *P*-value and % mean lifespan change are relative to *rrf-3(pk1426)* RNAi L4440 | | | | | | |
| Trial of *memo-1* knock down by RNAi starting at the first day of adulthood at 20°C (with FuDR) | | | | | | |
| wild type (N2) **L4440 (control)** | 23.1 ± 0.2 | 24 | 69/80 |  |  |  |
| wild type (N2) ***memo-1(RNAi#1)*** | 25.3 ± 0.3 | 26 | 84/93 | +10 | <0.0001 |  |
| wild type (N2) ***memo-1(RNAi#2)*** | 24.8 ± 0.3 | 26 | 62/73 | +7 | <0.0001 |  |
| *P*-value and % mean lifespan change are relative to wild type (N2) RNAi L4440 | | | | | | |
| Trial of *memo-1* and *skn-1* knock down by RNAi starting at the first day of adulthood at 20°C (with FuDR) | | | | | | |
| wild type (N2) **L4440 (control)** | 21.7 ± 0.6 | 27 | 94/106 |  |  | Fig. 2B, 2C |
| wild type (N2) ***memo-1(RNAi#1)*** | 26.3 ± 0.4 | 29 | 112/124 | +21 | <0.0001 | Fig. 2C |
| wild type (N2) ***skn-1(RNAi)**** | 16.4 ± 0.4 | 18 | 111/120 | -24 | <0.0001 | Fig. 2B |
| *memo-1(gk345)* **L4440** | 24.9 ± 0.3 | 27 | 159/166 | +15 | <0.0001 | Fig. 2B |
| *memo-1(gk345)* ***skn-1(RNAi)*** | 15.6 ± 0.3 | 18 | 114/122 | -28 | <0.0001, *<0.0788 | Fig. 2B |
| *skn-1(zu67)* **L4440** ^$^ | 12.4 ± 0.3 | 13 | 34/36 | -43 | <0.0001 | Fig. 2C |
| *skn-1(zu67)* ***memo-1(RNAi#1)*** | 13.2 ± 0.6 | 13 | 32/38 | -39 | <0.0001, ^$^0.6920 | Fig. 2C |
| *skn-1(zu129)* **L4440**^ | 11.7 ± 0.3 | 13 | 27/34 | -46 | <0.0001 |  |
| *skn-1(zu129)* ***memo-1(RNAi#1)*** | 12.4 ± 0.7 | 13 | 26/33 | -43 | <0.0001, ^0.5660 |  |
| *P*-value and % mean lifespan change are relative to wild type (N2) RNAi L4440, ***** wild type (N2) ***skn-1(RNAi),*** ^$^ *skn-1(zu67)* **L4440,** ^ *skn-1(zu129)* **L4440** | | | | | | |
| Trial of *skn-1* and *bli-3* knock down by RNAi starting at the first day of adulthood at 20°C (with FuDR) | | | | | | |
| wild type (N2) **L4440 (control)** | 22.7 ± 0.3 | 24 | 53/57 |  |  |  |
| wild type (N2) ***skn-1(RNAi)**** | 17.3 ± 0.4 | 19 | 67/73 | -24 | <0.0001 |  |
| wild type (N2) ***bli-3(RNAi)*** ^$^ | 21.8 ± 0.3 | 22 | 74/81 | -4 | 0.0528 |  |
| *memo-1(gk345)* **L4440**^ | 26.9 ± 0.5 | 29 | 56/60 | +19 | <0.0001 |  |
| *memo-1(gk345)* ***skn-1(RNAi)*** | 17.9 ± 0.5 | 19 | 47/54 | -21 | <0.0001, *<0.3591 |  |
| *memo-1(gk345)* ***bli-3(RNAi)*** | 21.5 ± 0.6 | 24 | 45/51 | -5 | 0.3513,  ^$^0.7744,  ^<0.0001 |  |
| *P*-value and % mean lifespan change are relative to wild type (N2) RNAi L4440, ***** wild type (N2) ***skn-1(RNAi),*** ^$^ wild type (N2) ***bli-3(RNAi)*,** ^ *memo-1(gk345)* **L4440** | | | | | | |
| Trial of *skn-1* and *bli-3* knock down by RNAi starting at the first day of adulthood at 20°C (with FuDR) | | | | | | |
| wild type (N2) **L4440 (control)** | 22.3 ± 0.2 | 23 | 143/161 |  |  |  |
| wild type (N2) ***skn-1(RNAi)**** | 16.2 ± 0.2 | 16 | 145/174 | -27 | <0.0001 |  |
| wild type (N2) ***bli-3(RNAi)*** ^$^ | 22.1 ± 0.2 | 23 | 131/153 | -1 | 0.3288 |  |
| *memo-1(gk345)* **L4440**^ | 27.2 ± 0.4 | 30 | 153/177 | +22 | <0.0001 |  |
| *memo-1(gk345)* ***skn-1(RNAi)*** | 16.9 ± 0.2 | 19 | 163/194 | -24 | <0.0001, *<0.061 |  |
| *memo-1(gk345)* ***bli-3(RNAi)*** | 22.0 ± 0.2 | 23 | 151/176 | -1 | 0.2564,  ^$^0.8021,  ^<0.0001 |  |
| *P*-value and % mean lifespan change are relative to wild type (N2) RNAi L4440, ***** wild type (N2) ***skn-1(RNAi),*** ^$^ wild type (N2) ***bli-3(RNAi)*,** ^ *memo-1(gk345)* **L4440** | | | | | | |
| Trial of *skn-1* and *bli-3* knock down by RNAi starting at the first day of adulthood at 20°C (with FuDR) | | | | | | |
| wild type (N2) **L4440 (control)** | 25.1 ± 0.4 | 28 | 85/95 |  |  | Fig. 4G |
| wild type (N2) ***skn-1(RNAi)**** | 18.0 ± 0.2 | 18 | 96/109 | -28 | <0.0001 |  |
| wild type (N2) ***bli-3(RNAi)*** ^$^ | 25.4 ± 0.3 | 28 | 113/126 | +1 | 0.5610 | Fig. 4G |
| *memo-1(gk345)* **L4440**^ | 30.0 ± 0.4 | 32 | 118/130 | +20 | <0.0001 | Fig. 4G |
| *memo-1(gk345)* ***skn-1(RNAi)*** | 18.5 ± 0.2 | 21 | 120/133 | -26 | <0.0001, *<0.0631 |  |
| *memo-1(gk345)* ***bli-3(RNAi)*** | 24.5 ± 0.4 | 28 | 104/119 | -2 | 0.4860,  ^$^0.2204,  ^<0.0001 | Fig. 4G |
| *P*-value and % mean lifespan change are relative to wild type (N2) RNAi L4440, ***** wild type (N2) ***skn-1(RNAi),*** ^$^ wild type (N2) ***bli-3(RNAi)*,** ^ *memo-1(gk345)* **L4440** | | | | | | |
| Trial of *sod-3* knock down by RNAi starting at the first day of adulthood at 20°C (with FuDR) | | | | | | |
| wild type (N2) **L4440 (control)** | 23.4 ± 0.6 | 25 | 60/74 |  |  |  |
| wild type (N2) ***sod-3(RNAi)**** | 22.2 ± 0.3 | 23 | 81/94 | -5 | 0.0232 |  |
| *memo-1(gk345)* **L4440**^ | 26.7 ± 0.4 | 30 | 88/98 | +14 | <0.0001 |  |
| *memo-1(gk345)* ***sod-3(RNAi)*** | 27.0 ± 0.3 | 28 | 94/105 | +15 | <0.0001, *<0.0001  ^<0.9036 |  |
| *P*-value and % mean lifespan change are relative to wild type (N2) RNAi L4440, ***** wild type (N2) ***sod-3(RNAi),*** ^ *memo-1(gk345)* **L4440** | | | | | | |
| Trial of *memo-1* knock down by RNAi starting at the first day of adulthood at 20°C (with FuDR) | | | | | | |
| wild type (N2) **L4440 (control)** | 22.6 ± 0.3 | 24 | 86/92 |  |  | Fig. 2H |
| wild type (N2) ***memo-1(RNAi#1)**** | 26.7 ± 0.4 | 29 | 95/102 | +18 | <0.0001 | Fig. 2H |
| *sek-1(km4)* **L4440**^$^ | 22.1 ± 0.6 | 26 | 55/62 | -2 | 0.8203 | Fig. 2H |
| *sek-1(km4)* ***memo-1(RNAi#1)*** | 21.9 ± 0.5 | 24 | 50/56 | -3 | 0.6685  *<0.0001,  ^$^0.6080 | Fig. 2H |
| *P*-value and % mean lifespan change are relative to wild type (N2) RNAi L4440, ***** wild type (N2) ***memo-1(RNAi#1),*** ^$^ *sek-1(km4)* **L4440** | | | | | | |
| Trial of *memo-1* knock down by RNAi starting at the first day of adulthood at 20°C (with FuDR) | | | | | | |
| wild type (N2) **L4440 (control)** | 25.4 ± 0.5 | 28 | 86/102 |  |  |  |
| wild type (N2) ***memo-1(RNAi#1)**** | 29.4 ± 0.5 | 32 | 76/89 | +16 | <0.0001 |  |
| *sek-1(km4)* **L4440**^$^ | 25.7 ± 0.5 | 30 | 67/80 | +1 | 0.3734 |  |
| *sek-1(km4)* ***memo-1(RNAi#1)*** | 26.6 ± 0.5 | 30 | 68/81 | +5 | 0.1381  *<0.0001,  ^$^0.2694 |  |
| *P*-value and % mean lifespan change are relative to wild type (N2) RNAi L4440, ***** wild type (N2) ***memo-1(RNAi#1),*** ^$^ *sek-1(km4)* **L4440** | | | | | | |
| Trial of *memo-1* knock down by RNAi with 5mM GSH treatment starting at the first day of adulthood at 20°C (with FuDR) | | | | | | |
| wild type (N2) **L4440 (control)** | 23.2 ± 0.2 | 24 | 104/119 |  |  | Fig. 3H |
| wild type (N2) **L4440 (5mM GSH)*** | 23.2 ± 0.2 | 26 | 118/134 | +0 | 0.7980 | Fig. 3H |
| wild type (N2) ***memo-1(RNAi#1)* (control)**” | 25.6 ± 0.4 | 26 | 90/107 | +10 | <0.0001 |  |
| wild type (N2) ***memo-1(RNAi#1)* (5mM GSH)**^$^ | 23.2 ± 0.3 | 24 | 116/129 | +0 | 0.5772  *0.7390  “<0.0001 |  |
| *memo-1(gk345)* **L4440(control)**^ | 28.9 ± 0.2 | 31 | 128/148 | +25 | <0.0001 | Fig. 3H |
| *memo-1(gk345)* **L4440(5mM GSH)** | 25.3 ± 0.2 | 26 | 116/126 | +9 | <0.0001  *<0.0001  ^<0.0001 | Fig. 3H |
| *P*-value and % mean lifespan change are relative to wild type (N2) RNAi L4440, ***** wild type (N2) **L4440 (5mM GSH)*,* “** wild type (N2) ***memo-1(RNAi#1)* (control),** ^$^ wild type (N2) ***memo-1(RNAi#1)* (5mM GSH),** ^ *memo-1(gk345)* **L4440(control)** | | | | | | |
| Trial of *memo-1* knock down by RNAi with 5mM GSH treatment starting at the first day of adulthood at 20°C (with FuDR) | | | | | | |
| wild type (N2) **L4440 (control)** | 25.0 ± 0.5 | 28 | 42/48 |  |  |  |
| wild type (N2) **L4440 (5mM GSH)*** | 24.9 ± 0.5 | 28 | 32/41 | -0 | 0.4482 |  |
| wild type (N2) ***memo-1(RNAi#1)* (control)**” | 27.1 ± 0.4 | 31 | 49/56 | +8 | 0.0451 |  |
| wild type (N2) ***memo-1(RNAi#1)* (5mM GSH)** | 24.2 ± 0.3 | 28 | 37/43 | -3 | 0.0524  *0.3586  “<0.0001 |  |
| *P*-value and % mean lifespan change are relative to wild type (N2) RNAi L4440, ***** wild type (N2) **L4440 (5mM GSH)*,* “** wild type (N2) ***memo-1(RNAi#1)* (control)** | | | | | | |
| Trial of BLI-3/NADPH oxidase overexpression at 20°C (with FuDR) | | | | | | |
| wild type (N2) | 22.2 ± 0.2 | 24 | 94/103 |  |  |  |
| *ldEx102* [*rol-6(su1006)*] control * | 22.2 ± 0.3 | 24 | 92/104 | +0 | 0.9345 |  |
| *ldEx117* [**BLI-3**; *rol-6(su1006)*] | 23.1 ± 0.4 | 26 | 72/81 | +4 | 0.0085  *0.1435 |  |
| *P*-value and % mean lifespan change are relative to wild type (N2) and ***** *ldEx102* [*rol-6(su1006)*] control | | | | | | |
| Trial of BLI-3/NADPH oxidase and maturation factors DOXA-1 and TSP-15 triple overexpression at 20°C (with FuDR) | | | | | | |
| non-transgenic siblings | 21.5 ± 0.2 | 23 | 72/82 |  |  |  |
| *ldEx118* [**BLI-3, DOXA-1, TSP-15**; *rol-6(su1006)*] | 25.4 ± 0.4 | 28 | 77/89 | +18 | <0.0001 |  |
| *P*-value and % mean lifespan change are relative to non-transgenic siblings | | | | | | |
| Trial of BLI-3/NADPH oxidase and maturation factors DOXA-1 and TSP-15 triple overexpression at 20°C (with FuDR) | | | | | | |
| wild type (N2) | 21.5 ± 0.3 | 22 | 80/91 |  |  |  |
| *ldEx102* [*rol-6(su1006)*] control * | 22.3 ± 0.3 | 25 | 99/107 | +4 | 0.0404 |  |
| *ldEx118* [**BLI-3, DOXA-1, TSP-15**; *rol-6(su1006)*] | 25.9 ± 0.4 | 29 | 114/124 | +20 | <0.0001  *<0.0001 |  |
| *P*-value and % mean lifespan change are relative to wild type (N2) and ***** *ldEx102* [*rol-6(su1006)*] control | | | | | | |
| Trial of BLI-3/NADPH oxidase and maturation factors DOXA-1 and TSP-15 triple overexpression at 20°C (with FuDR) | | | | | | |
| wild type (N2) | 21.6 ± 0.2 | 23 | 56/68 |  |  |  |
| *ldEx102* [*rol-6(su1006)*] control * | 21.9 ± 0.3 | 23 | 69/81 | +1 | 0.3926 |  |
| *ldEx117* [**BLI-3**; *rol-6(su1006)*] | 24.5 ± 0.5 | 28 | 111/125 | +13 | 0.0002  *0.0002 |  |
| *ldEx118* [**BLI-3, DOXA-1, TSP-15**; *rol-6(su1006)*] | 23.3 ± 0.3 | 23 | 94/104 | +8 | <0.0001  *<0.0001 |  |
| *P*-value and % mean lifespan change are relative to wild type (N2) and ***** *ldEx102* [*rol-6(su1006)*] control | | | | | | |
| Trial of BLI-3/NADPH oxidase and maturation factors DOXA-1 and TSP-15 triple overexpression at 20°C (with FuDR) | | | | | | |
| wild type (N2) | 20.8 ± 0.3 | 23 | 61/70 |  |  |  |
| *ldEx118* [**BLI-3, DOXA-1, TSP-15**; *rol-6(su1006)*] | 26.5 ± 0.5 | 29 | 63/72 | +27 | <0.0001 |  |
| *memo-1(gk345)* | 25.7 ± 0.4 | 29 | 71/83 | +23 | <0.0001 |  |
| *P*-value and % mean lifespan change are relative to wild type (N2) | | | | | | |
| Trial of BLI-3/NADPH oxidase and maturation factors DOXA-1 and TSP-15 triple overexpression at 20°C (with FuDR) | | | | | | |
| *ldEx102* [*rol-6(su1006)*] **L4440** | 20.2 ± 0.6 | 23 | 60/65 |  |  | Fig. 4J |
| *ldEx102* [*rol-6(su1006)*] ***bli-3(RNAi)**** | 19.1 ± 0.5 | 23 | 61/75 | -5 | 0.2632 | Fig.4K |
| *ldEx102* [*rol-6(su1006)*] ***skn-1(RNAi)***^$^ | 17.1 ± 0.3 | 18 | 101/112 | -15 | <0.0001 | Fig.4L |
| *ldEx102* [*rol-6(su1006)*] **L4440 5 mM GSH^@^** | 20.0 ± 0.8 | 25 | 52/55 | -1 | 0.8791 | Fig.4M |
| *ldEx118* [**BLI-3, DOXA-1, TSP-15**; *rol-6(su1006)*]  **L4440“** | 23.2 ± 0.5 | 25 | 92/112 | +15 | <0.0001 | Fig.4J |
| *ldEx118* [**BLI-3, DOXA-1, TSP-15**; *rol-6(su1006)*]  ***bli-3(RNAi)*** | 19.7 ± 0.6 | 23 | 70/77 | -2 | 0.6383  *0.7997 | Fig.4K |
| *ldEx118* [**BLI-3, DOXA-1, TSP-15**; *rol-6(su1006)*]  ***skn-1(RNAi)*** | 17.7 ± 0.4 | 21 | 101/109 | -12 | <0.0001  ^$^0.0765 | Fig.4L |
| *ldEx118* [**BLI-3, DOXA-1, TSP-15**; *rol-6(su1006)*]  **L4440 5 mM GSH** | 20.5 ± 0.5 | 25 | 66/75 | +1 | 0.4021  ^@^0.3558  “<0.0208 | Fig.4M |
| *P*-value and % mean lifespan change are relative to *ldEx102* [*rol-6(su1006)*] **L4440,** *ldEx102* [*rol-6(su1006)*] ***bli-3(RNAi)*,*** *ldEx102* [*rol-6(su1006)*] ***skn-1(RNAi)***^$^*, ldEx102* [*rol-6(su1006)*] **L4440 5 mM GSH^@^,** *ldEx118* [**BLI-3, DOXA-1, TSP-15**; *rol-6(su1006)*]  **L4440“** | | | | | | |
| Trial of BLI-3/NADPH oxidase and maturation factors DOXA-1 and TSP-15 triple overexpression at 20°C (with FuDR) | | | | | | |
| *ldEx102* [*rol-6(su1006)*] **(control)** | 20.5 ± 0.5 | 23 | 90/111 |  |  |  |
| *ldEx102* [*rol-6(su1006)*] **5 mM GSH*** | 21.1 ± 0.5 | 23 | 61/69 | +3 | 0.5470 |  |
| *ldEx118* [**BLI-3, DOXA-1, TSP-15**; *rol-6(su1006)*]  **(control)”** | 22.8 ± 0.6 | 28 | 100/110 | +11 | 0.0008 |  |
| *ldEx118* [**BLI-3, DOXA-1, TSP-15**; *rol-6(su1006)*]  **5 mM GSH** | 21.3 ± 0.6 | 28 | 83/94 | +4 | 0.0403  *0.5900  “0.0521 |  |
| *P*-value and % mean lifespan change are relative to *ldEx102* [*rol-6(su1006)*] **(control),** *ldEx102* [*rol-6(su1006)*] **5 mM GSH*,** *ldEx118* [**BLI-3, DOXA-1, TSP-15**; *rol-6(su1006)*]  **(control)”** | | | | | | |
| Trial of BLI-3/NADPH oxidase and maturation factors DOXA-1 and TSP-15 triple overexpression at 20°C (with FuDR) | | | | | | |
| wild type (N2) | 21.4 ± 0.5 | 23 | 52/56 |  |  |  |
| *memo-1(gk345)* | 25.4 ± 0.6 | 28 | 86/95 | +18 | <0.0001 |  |
| *ldEx102* [*rol-6(su1006)*]* | 22.1 ± 0.3 | 23 | 63/71 | +3 | 0.3678 |  |
| *ldEx117* [**BLI-3**; *rol-6(su1006)*] | 22.0 ± 0.4 | 23 | 73/78 | +3 | 0.5100  *0.9266 |  |
| *ldEx118* [**BLI-3, DOXA-1, TSP-15**; *rol-6(su1006)*] | 26.4 ± 0.7 | 30 | 91/93 | +23 | <0.0001  *<0.0001 |  |
| *P*-value and % mean lifespan change are relative to wild type (N2) and ***** *ldEx102* [*rol-6(su1006)*] | | | | | | |
| Trial of BLI-3/NADPH oxidase and maturation factors DOXA-1 and TSP-15 triple overexpression at 20°C (with FuDR) | | | | | | |
| wild type (N2) | 22.8 ± 0.7 | 25 | 36/46 |  |  |  |
| *ldEx102* [*rol-6(su1006)*]* | 23.7 ± 0.4 | 25 | 75/90 | +4 | 0.4209 |  |
| *ldEx118* [**BLI-3, DOXA-1, TSP-15**; *rol-6(su1006)*] | 26.1 ± 0.7 | 30 | 69/73 | +25 | <0.0001  *0.0007 |  |
| *P*-value and % mean lifespan change are relative to wild type (N2) and ***** *ldEx102* [*rol-6(su1006)*] | | | | | | |
| Trial of *rho-1* knock down by RNAi starting at the first day of adulthood at 20°C (with FuDR) | | | | | | |
| wild type (N2) **L4440 (control)** | 24.3 ± 0.3 | 27 | 149/163 |  |  | Fig. 5D |
| wild type (N2) ***rho-1(RNAi)**** | 21.9 ± 0.2 | 25 | 104/124 | -10 | <0.0001 | Fig. 5D |
| *memo-1(gk345)* **L4440**^ | 27.7 ± 0.4 | 32 | 148/164 | +14 | <0.0001 | Fig. 5D |
| *memo-1(gk345)* ***rho-1(RNAi)*** | 22.3 ± 0.2 | 25 | 104/132 | -8 | <0.0001, *<0.4460  ^<0.0001 | Fig. 5D |
| *P*-value and % mean lifespan change are relative to wild type (N2) RNAi L4440, ***** wild type (N2) ***rho-1(RNAi),*** ^ *memo-1(gk345)* **L4440** | | | | | | |
| Trial of *rho-1* knock down by RNAi starting at the first day of adulthood at 20°C (with FuDR) | | | | | | |
| wild type (N2) **L4440 (control)** | 22.7 ± 0.5 | 25 | 73/91 |  |  | Fig. 5E |
| wild type (N2) ***rho-1(RNAi)**** | 19.9 ± 0.3 | 23 | 74/106 | -12 | <0.0001 | Fig. 5E |
| *memo-1(gk345)* **L4440**^ | 27.4 ± 0.5 | 30 | 107/128 | +21 | <0.0001 |  |
| *memo-1(gk345)* ***rho-1(RNAi)*** | 20.3 ± 0.3 | 23 | 125/161 | -11 | <0.0001, *<0.3596  ^<0.0001, |  |
| *ldEx118* [**BLI-3, DOXA-1, TSP-15**; *rol-6(su1006)*]  **L4440**” | 28.1 ± 0.5 | 32 | 91/105 | +24 | <0.0001 | Fig. 5E |
| *ldEx118* [**BLI-3, DOXA-1, TSP-15**; *rol-6(su1006)*]  ***rho-1(RNAi)*** | 24.6 ± 0.4 | 28 | 91/104 | +8 | <0.0044, *<0. 0001,  “<0.0001 | Fig. 5E |
| *P*-value and % mean lifespan change are relative to wild type (N2) RNAi L4440, ***** wild type (N2) ***rho-1(RNAi),*** ^ *memo-1(gk345)* **L4440,** *ldEx118* [**BLI-3, DOXA-1, TSP-15**; *rol-6(su1006)*]  **L4440**” | | | | | | |
| Trial of *bli-3* and *memo-1* knock down by RNAi starting at the first day of adulthood at 20°C (with FuDR) | | | | | | |
| wild type **L4440 (control)** | 24.8 ± 0.4 | 28 | 114/124 |  |  |  |
| wild type ***bli-3(RNAi)**** | 23.8 ± 0.3 | 25 | 100/126 | -4 | 0.0442 |  |
| wild type ***memo-1(RNAi#1)*** | 28.1 ± 0.4 | 30 | 137/148 | +13 | <0.0001 |  |
| *isp-1(qm150)* **L4440**^$^ | 29.4 ± 0.5 | 32 | 92/107 | +19 | <0.0001 |  |
| *isp-1(qm150)* ***bli-3(RNAi)*** | 28.8 ± 0.4 | 30 | 134/155 | +16 | <0.0001  *<0.0001,  ^$^0.1414 |  |
| *isp-1(qm150)* ***memo-1(RNAi#1)*** | 32.1 ± 0.4 | 35 | 109/125 | +29 | <0.0001,  ^$^<0.0001 |  |
| *P*-value and % mean lifespan change are relative to wild type **L4440**, ***** wild type ***bli-3(RNAi),*** ^$^ *isp-1(qm150)* **L4440** | | | | | | |
| Trial of *bli-3* knock down by RNAi starting at the first day of adulthood at 20°C (with FuDR) | | | | | | |
| *rrf-3(pk1426)* **L4440 (control)** | 21.3 ± 0.6 | 24 | 65/75 |  |  |  |
| *rrf-3(pk1426)* ***bli-3(RNAi)**** | 20.9 ± 0.5 | 24 | 95/101 | -2 | 0.4683 |  |
| *rrf-3(pk1426);* *daf-2(e1370)* **L4440**^$^ | 43.6 ± 1.1 | 50 | 79/82 | +105 | <0.0001 |  |
| *rrf-3(pk1426);* *daf-2(e1370)* ***bli-3(RNAi)*** | 41.0 ± 1.1 | 50 | 86/89 | +92 | <0.0001  *<0.0001,  ^$^0.0945 |  |
| *P*-value and % mean lifespan change are relative to *rrf-3(pk1426)* **L4440**, ***** *rrf-3(pk1426)* ***bli-3(RNAi),*** ^$^ *rrf-3(pk1426);* *daf-2(e1370)* **L4440** | | | | | | |
| Trial of *bli-3* knock down by RNAi starting at the first day of adulthood at 20°C (with FuDR) | | | | | | |
| *rrf-3(pk1426)* **L4440 (control)** | 21.4 ± 0.3 | 22 | 85/105 |  |  |  |
| *rrf-3(pk1426)* ***bli-3(RNAi)**** | 19.9 ± 0.2 | 22 | 98/106 | -7 | 0.0011 |  |
| *rrf-3(pk1426)* *eat-2(ad1116)* **L4440**^$^ | 30.6 ± 0.9 | 36 | 69/76 | +43 | <0.0001 |  |
| *rrf-3(pk1426)* *eat-2(ad1116)* ***bli-3(RNAi)*** | 28.0 ± 0.9 | 34 | 81/85 | +31 | <0.0001  *<0.0001,  ^$^0.1454 |  |
| *P*-value and % mean lifespan change are relative to *rrf-3(pk1426)* **L4440**, ***** *rrf-3(pk1426)* ***bli-3(RNAi),*** ^$^ *rrf-3(pk1426)* *eat-2(ad1116)* **L4440** | | | | | | |

Trials that were performed in parallel are grouped together. (N) = number of animals observed; Lifespan was measured from the L4 stage (see Materials and Methods for details). Animals that left the plates, buried into the agar, bagged, or exploded were censored. L4440 empty vector was otherwise used as the control.
