# Supplementary material for: NADPH oxidase-mediated redox signaling promotes oxidative stress resistance and longevity through memo-1 in C. elegans
Source: eLife. 2017 Jan 13;6:e19493. doi: 10.7554/eLife.19493 (PMC5235354; doi:10.7554/eLife.19493)
Supplement: Supplementary file 2. — DOI: http://dx.doi.org/10.7554/eLife.19493.016 [file elife-19493-supp2.docx]

**Supplementary File 2. Loss of *memo-1* increases oxidative stress resistance**

| Strain / **RNAi** | Mean lifespan ± S.E.M.  [hours] | 75^th^ percentile  [hours] | N dead/ Initial N | % mean survival change to control | P-value (log-rank) vs. control | Figure |
| --- | --- | --- | --- | --- | --- | --- |
| **Trials of day 1 adults treated with arsenite** | | | | | | |
| Trial of *memo-1(gk345)* mutants at 20°C (5 mM arsenite) | | | | | | |
| wild type (N2) | 17.2 ± 1.6 | 26 | 36/37 |  |  | Fig. 1K |
| *memo-1(gk345)* mutants | 25.2 ± 2.3 | 32 | 40/41 | +47 | 0.0022 | Fig. 1K |
| P-value and % mean lifespan change are relative to wild type (N2) | | | | | | |
| Trial of *memo-1(gk345)* mutants at 20°C (5 mM arsenite) | | | | | | |
| wild type (N2) | 21.3 ± 1.9 | 28 | 36/38 |  |  |  |
| *memo-1(gk345)* mutants | 30.4 ± 1.6 | 33 | 39/43 | +43 | 0.0014 |  |
| P-value and % mean lifespan change are relative to wild type (N2) | | | | | | |
| Trial of *memo-1(gk345)* mutants at 20°C (5 mM arsenite) | | | | | | |
| wild type (N2) | 22.8 ± 1.4 | 27 | 42/42 |  |  |  |
| *memo-1(gk345)* mutants | 26.3 ± 1.6 | 38 | 50/50 | +15 | 0.0188 |  |
| P-value and % mean lifespan change are relative to wild type (N2) | | | | | | |
| Trial of *memo-1(gk345)* mutants at 20°C (5 mM arsenite) | | | | | | |
| wild type (N2) | 17.9 ± 0.3 | 23 | 79/79 |  |  |  |
| *memo-1(gk345)* mutants | 20.8 ± 0.3 | 25 | 89/89 | +16 | <0.0001 |  |
| P-value and % mean lifespan change are relative to wild type (N2) | | | | | | |
| Trial of *memo-1(gk345)* mutants at 20°C (10 mM arsenite) | | | | | | |
| wild type (N2) | 9.2 ± 0.8 | 12 | 34/38 |  |  |  |
| *memo-1(gk345)* mutants | 12.3 ± 1.2 | 21 | 33/34 | +34 | 0.0070 |  |
| P-value and % mean lifespan change are relative to wild type (N2) | | | | | | |
| Trial of *memo-1* knock down by RNAi for two generations at 20°C (5 mM arsenite) | | | | | | |
| wild type (N2) RNAi **L4440 (control)** | 20.8 ± 1.6 | 28 | 36/36 |  |  |  |
| wild type (N2) RNAi ***memo-1(RNAi#1)*** | 28.9 ± 1.4 | 30 | 43/44 | +39 | 0.0001 |  |
| wild type (N2) RNAi ***memo-1(RNAi#2)*** | 28.8 ± 2.0 | 33 | 40/41 | +38 | 0.0001 |  |
| P-value and % mean lifespan change are relative to wild type (N2) RNAi L4440 | | | | | | |
| Trial of *memo-1* knock down by RNAi for two generations at 20°C (5 mM arsenite) | | | | | | |
| wild type (N2) RNAi **L4440 (control)** | 16.7 ± 0.6 | 18 | 48/48 |  |  |  |
| wild type (N2) RNAi ***memo-1(RNAi#1)*** | 22.5 ± 1.4 | 36 | 53/53 | +35 | 0.0002 |  |
| wild type (N2) RNAi ***memo-1(RNAi#2)*** | 27.4 ± 1.5 | 41 | 60/67 | +64 | <0.0001 |  |
| P-value and % mean lifespan change are relative to wild type (N2) RNAi L4440 | | | | | | |
| Trial of double RNAi knock down of *memo-1* and *rho-1* for one generations at 20°C (5 mM arsenite) | | | | | | |
| wild type (N2) RNAi **L4440/L4440 (control)** | 11.9 ± 0.5 | 14 | 26/27 |  |  | Fig. 5B |
| wild type (N2) RNAi ***memo-1(RNAi#1)/*L4440** | 14.4 ± 0.6 | 16 | 33/33 | +21 | 0.0019 | Fig. 5B |
| wild type (N2) RNAi ***rho-1*/L4440** | 12.0 ± 0.6 | 14 | 16/16 | +1 | 0.9651 | Fig. 5B |
| wild type (N2) RNAi ***memo-1(RNAi#1)/rho-1*** | 10.8 ± 0.3 | 12 | 22/27 | -9 | 0.0866 | Fig. 5B |
| P-value and % mean lifespan change are relative to wild type (N2) RNAi L4440 | | | | | | |
| Trial of *memo-1* knock down by RNAi for one generations at 20°C (5 mM arsenite) | | | | | | |
| wild type (N2) RNAi **L4440 (control)** | 12.2 ± 0.4 | 14 | 32/34 |  |  | Fig. 3F |
| wild type (N2) RNAi ***memo-1(RNAi#1)*** | 15.4 ± 0.6 | 18 | 43/45 | +26 | <0.0001 | Fig. 3F |
| *sek-1(km4)* mutants RNAi **L4440** | 11.1 ± 0.4 | 12 | 27/27 | -9 | 0.0861 | Fig. 3F |
| *sek-1(km4)* mutants RNAi ***memo-1(RNAi#1)*** | 11.6 ± 0.4 | 14 | 35/35 | -5 | 0.2376 | Fig. 3F |
| P-value and % mean lifespan change are relative to wild type (N2) RNAi L4440 | | | | | | |
|  | | | | | | |
| **Trials of day 3 adults treated with tert-butyl hydrogen peroxide (t-BOOH).** | | | | | | |
| Trial of *memo-1(gk345)* mutants at 20°C (15 mM t-BOOH) | | | | | | |
| wild type (N2) | 2.8 ± 0.2 | 3 | 16/24 |  |  |  |
| *memo-1(gk345)* mutants | 3.4 ± 0.2 | 4 | 25/31 | +21 | 0.0095 |  |
| P-value and % mean lifespan change are relative to wild type (N2) | | | | | | |
| Trial of *memo-1(gk345)* mutants at 20°C (15 mM t-BOOH) | | | | | | |
| wild type (N2) | 3.5 ± 0.3 | 4 | 25/39 |  |  |  |
| *memo-1(gk345)* mutants | 4.5 ± 0.4 | 4 | 14/33 | +29 | 0.0459 |  |
| P-value and % mean lifespan change are relative to wild type (N2) | | | | | | |
| Trial of *memo-1* knock down by RNAi for two generations at 20°C (15 mM t-BOOH) | | | | | | |
| wild type (N2) RNAi **L4440 (control)** | 5.4 ± 0.4 | 7 | 31/31 |  |  |  |
| wild type (N2) RNAi ***memo-1(RNAi#1)*** | 6.3 ± 0.5 | 7 | 31/40 | +17 | 0.0375 |  |
| P-value and % mean lifespan change are relative to wild type (N2) RNAi L4440 | | | | | | |

Trials that were performed in parallel are grouped together. (N) = number of animals observed. The metalloid sodium arsenite attacks thiol groups on proteins and induces ROS. Tert-butyl hydrogen peroxide (t-BOOH) is an organic peroxide more stable than hydrogen peroxide in cells and induces ROS.For arsenite: Animals were censored when injured by transfer into physiological buffer (M9) containing sodium arsenite or when exploded. For t-BOOH: Animals were censored that left the plates, buried into the agar, or exploded. To escape the t-BOOH, most censored animals run off the agar containing the t-BOOH to the side plate. L4440 empty vector was otherwise used as the control. *memo-1(RNAi)* contract #1 is from Vidal library and #2 is from Ahringer library.
