# Supplementary material for: NADPH oxidase-mediated redox signaling promotes oxidative stress resistance and longevity through memo-1 in C. elegans
Source: eLife. 2017 Jan 13;6:e19493. doi: 10.7554/eLife.19493 (PMC5235354; doi:10.7554/eLife.19493)
Supplement: Supplementary file 3. — DOI: http://dx.doi.org/10.7554/eLife.19493.017 [file elife-19493-supp3.docx]

**Supplementary File 3. Primer Sequences**

| **Name** | **Sequence** |
| --- | --- |
| Primers for *memo-1(gk345)* deletion | |
| Memo1: | 5’-ATT TCC ATA CGT TTG CTG CC-3’ |
| Memo2: | 5’-CAT GCA TCC TCA TCC CTC TT-3’ |
| Memo3: | 5’-AAA TCG AAA TGA CGA GTG GC-3’ |
| Memo4: | 5’-GAC TGA AGA CTA CTC GCG GC-3’ |
| Primers for P*memo-1*::GFP fusion PCR | |
| memo-GFP-3rev: | 5’-GGA GGG TCT AGC TTT AAA GAT TTC TGT ATA TTA-3’ |
| memo-GFP-3for: | 5’-CCT TTT TTT TTT GGC GCC GAC GTC ACA TCC CGC CAG TCC TTC ACT CTT TT-3’ |
| memo-GFP-5for: | 5’TGA AAA GTT CTT CTC CTT TAC TCA TTT TAA ATT CAG ATG GTC TGA ATA TT-3’ |
| memo-GFP-5rev: | 5’-CGG AGA CTA TTT AAG GTC TTC CAA T-3’ |
| gfp-memo-for: | 5’-AAT ATT CAG ACC ATC TGA ATT TAA AAT GAG TAA AGG AGA AGA ACT TTT CA-3’ |
| gfp-memo-rev: | 5’-AAA AGA GTG AAG GAC TGG CGG GAT GTG ACG TCG GCG CCA AAA AAA AAA GG-3’ |
| memo-GFP-3rev-int: | 5’-GTT TAT TTG CCA ATC ACA ATA AAG TTG G-3’ |
| memo-GFP-5rev-int: | 5’-TTC TCC GAA CTC CTT CAT CTT GGA AGC-3’ |
| GFPintmemo-seq1: | 5’-TTC TAT TAA CAA GGG TAT CAC CTT C-3’ |
| GFPintmemo-seq2: | 5’-GTC GAT CAT CCG GAT TAC TTG TAT G-3’ |
